# Examining the impact of globalization and natural resources on environmental sustainability in G20 countries

**DOI:** 10.1038/s41598-024-81613-6

**Published:** 2024-12-28

**Authors:** Caihua Wang, Hamid Mahmood, Samia Khalid

**Affiliations:** 1 School of Marxism, Hebei Sport University, Shijiazhuang, 050041 Hebei P. R. China; 2 School of Economics and Finance, Xi’an Jiaotong University, Xi’an, Shaanxi P.R. China

**Keywords:** Environmental sustainability, Globalization, Natural resources, CUP-FM & CUP-BC, Environmental social sciences, Environmental economics, Environmental impact

## Abstract

The G20 countries are responsible for around 75% of the world’s greenhouse gas (GHG) emissions, including the use of natural resources. In this regard, the role of globalization in achieving environmental sustainability is a relatively new topic of concern. As a result, the present study considers how globalization and natural resources affect GHG emissions, as well as the roles that renewable energy consumption and urbanization play in the G20 countries between 1990 and 2020. Moreover, this research utilizes advanced panel data estimation methods to assess these interrelationships, like CUP-FM and CUP-BC methods. The study finds that globalization and natural resource use contribute to increased GHG emissions, with a more pronounced effect in high-income countries because most of the countries have extended carbon taxes or emissions trading systems in the last few years. Conversely, the adoption of renewable energy is associated with a reduction in emissions, underscoring the importance of transitioning to cleaner energy sources. Urbanization trends also correlate positively with GHG emissions, highlighting the environmental impact of rapid urban growth, particularly in middle-income countries. These results underscore the need for targeted policy interventions. High-income countries should intensify efforts to reduce emissions through renewable energy and stricter regulations on resource consumption. While, middle-income countries require support to manage urbanization and adopt sustainable practices. This study provides crucial insights into the complex dynamics of globalization, resource use, and sustainability, offering valuable guidance for policymakers and researchers engaged in environmental management and climate change mitigation.

**Supplementary Information:**

The online version contains supplementary material available at 10.1038/s41598-024-81613-6.

## Introduction

The task of combining economic development with environmental sustainability is becoming increasingly pressing as globalization accelerates. Enhanced communication, technological advancements, and innovation have driven unprecedented economic expansion, yet this progress has also resulted in significant environmental impacts, including heightened greenhouse gas (GHG) emissions^1–3^. The debate over how to balance these competing priorities remains central to policy discussions, particularly as the environmental consequences of globalization become more pronounced^4,5^. Comprehending the intricate correlation between globalization (GN) and greenhouse gas emissions is imperative for proficient policy formulation.

While globalization can foster economic growth and technological advancement, which may ultimately reduce environmental impacts, it can also lead to increased emissions due to heightened industrial activity and energy consumption^6,7^. Effective policy responses to the immediate and long-term environmental implications of globalization, especially in high- and middle-income nations, need thorough analysis.

This study is essential because it tackles a crucial policy-level issue: how to balance globalization’s environmental effects with the pursuit of economic expansion. Specifically, it examines how GN, natural resources (NR), and renewable energy (RE) usage affect GHG emissions in the G20 countries. The G20, which represents 75% of global GHG emissions, has made substantial commitments to reduce emissions and achieve net-zero targets by 2050 (OECD, 2020). However, actual progress varies, with high-income countries making limited reductions and middle-income countries experiencing emission increases consistent with their targets^8^. This variation underscores the need for targeted, evidence-based policy recommendations that address the unique challenges faced by different income groups.

**Fig. 1:**
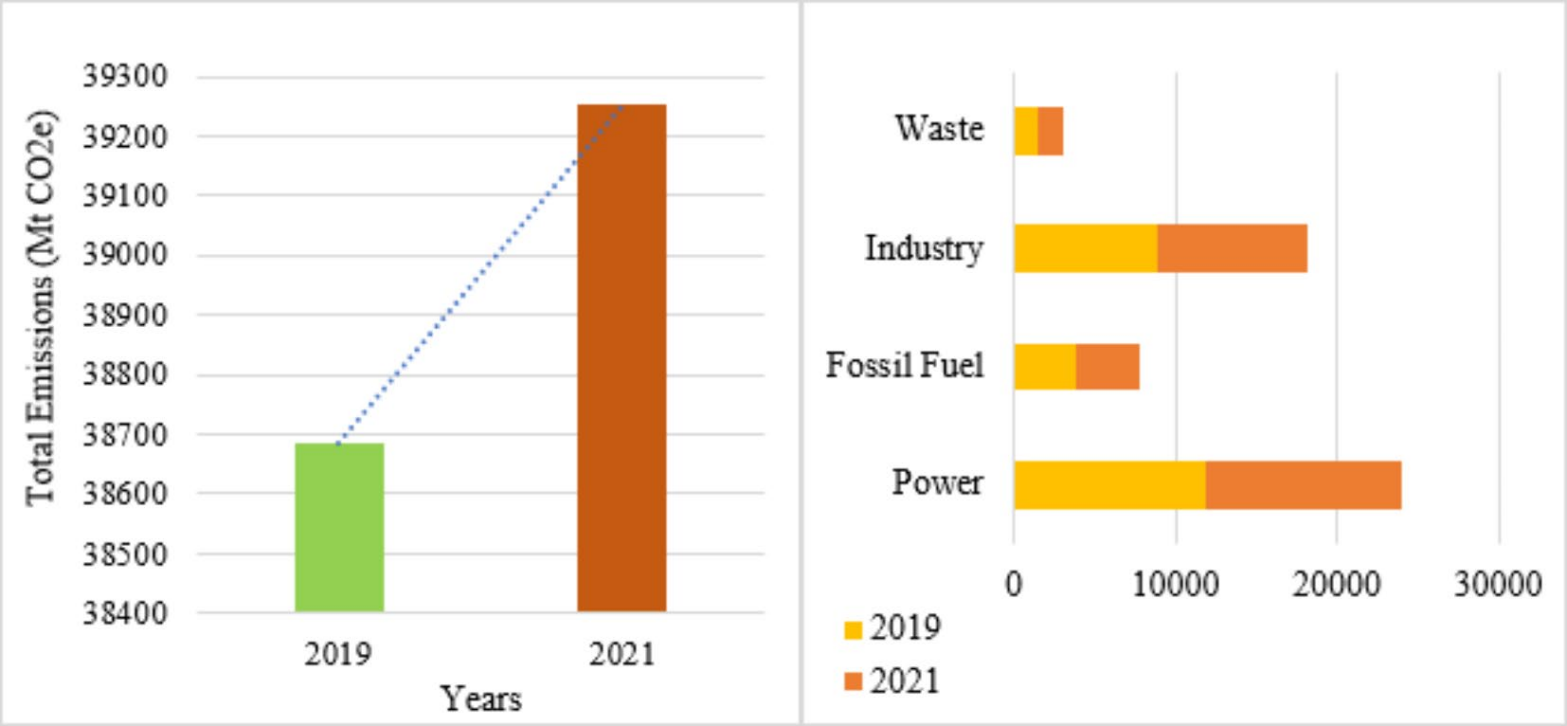
(**a**) Total emissions in G20 countries and Fig. 1(**b**) Sector-wise GHG emissions in G20 countries.

The rise in GHG emissions in G20 countries from 2019 to 2021 is a multifaceted issue driven by economic, industrial, and policy factors as shown in Fig. 1(a). As economies began to recover from the COVID-19 pandemic, industrial activities and energy consumption surged, leading to higher emissions. With the resumption of economic activities, there was a significant increase in energy demand^9^. As many G20 countries still rely heavily on fossil fuels for energy, leading to increased CO2 emissions. Moreover, in some countries, stimulus packages and economic incentives often focused on traditional industries, which are typically higher emitters. Another reason is that due to increased deforestation and changes in land use, often driven by agricultural expansion and urbanization, contributed to higher emissions^10^. In this sense, a persistent reliance on energy sources with high emissions might result from uneven and occasionally insufficient legislative initiatives to reduce emissions.

The Fig. 1(b) shows that both the power and industry sectors play a crucial role in the total GHG emissions of G20 countries. The power sector’s heavy reliance on fossil fuels and slow transition to renewable energy sources, combined with the industry sector’s energy-intensive processes and increased production, significantly contribute to the overall emissions^11,12^. The power sector in many G20 countries still relies heavily on coal, oil, and natural gas for electricity generation. Industries such as steel, cement, and chemicals are particularly energy-intensive and significant sources of GHG emissions. These industries rely heavily on processes that emit large amounts of CO2, such as the combustion of fossil fuels and chemical reactions in production processes^13^. For the G20 countries to accomplish their climate objectives and lessen their environmental effect, they must address emissions in these areas. To reduce emissions from these vital industries, comprehensive plans incorporating regulatory adjustments, technology developments, and investments in renewable energy and energy efficiency are required^14^.

Intensifying cross-border economic activity promotes development and technology interchange, but it also raises carbon emissions. For G20 countries—key global economic players—this means that their industrial processes, transportation networks, and consumption patterns often have significant environmental impacts^15^. The global demand for resources, such as oil, gas, and minerals, often leads to environmental degradation and higher emissions. As these countries are engage in global trade, they not only import and export goods but also the associated carbon footprint of their production. The reliance on fossil fuels in many G20 economies exacerbates this issue, as fossil fuel combustion is a major source of greenhouse gases. Industries in these countries contribute to global emissions through both domestic operations and by supplying goods to other nations. Additionally, the economic incentives to maximize production and consumption in a globalized economy can lead to inefficient resource use and greater carbon outputs. To address these challenges, G20 countries are increasingly focusing on policies aimed at reducing emissions. This includes investing in renewable energy technologies, enhancing energy efficiency, implementing carbon pricing mechanisms, and supporting international climate agreements such as the Paris Agreement^16^. In order to transition to a low-carbon economy that can support global growth while reducing the negative consequences of climate change, it is important to strike a balance between environmental stewardship and economic development^17^.

The study aims to provide actionable solutions by analyzing the effects of GN, NR, and RE on GHG emissions using a novel GN index that incorporates economic, social, and political dimensions. This approach offers a more nuanced understanding of globalization’s impact compared to traditional measures like trade openness or export/import ratios. By utilizing panel data cointegration and accounting for cross-country heterogeneity, the study provides robust long-term estimates of the interactions between globalization and emissions. The choice of the G20 countries as the sample is particularly relevant because they account for a substantial portion of global emissions and are central to international climate agreements. The results and policy recommendations derived from this research are expected to be generalizable to other large economies with similar profiles and can inform global and national climate strategies. Unlike previous studies that have primarily used trade indicators or aggregate measures of globalization, this research introduces a refined GN index and employs advanced econometric techniques to assess long-term trends and causal relationships. This distinction enhances the study’s contribution to the field by providing a clearer picture of how globalization influences emissions and by offering targeted policy recommendations that can help balance economic and environmental goals. In summary, this study fills a critical gap by integrating GN, NR, and REC into the analysis of GHG emissions and offering practical policy solutions. It tackles the urgent need for practical approaches to mitigate the effects of globalization on the environment, especially in the framework of the G20. The results will not only add to the body of knowledge in academia but will also provide guidance to policymakers to accomplish sustainable development goals.

The rest of the study consists of the following sections: An overview of pertinent research is given in Sect. Literature review, followed by the data and methodological framework in Sect. Theoretical framework. The empirical results and discussion are presented in Sect. Empirical findings and Sect. Discussion of the results. The last section concludes the study with policy recommendations for G20 countries.

## Literature review

In the existing literature, environmental sustainability has gotten a lot of attention, but there is still a lot of disagreement about it. The researchers investigated and reported on a wide range of environmental quality factors that might contribute to a more sustainable environment^4,5,18–22^. But GN, NR, and RE consumption are some of the recent factors that have received a lot of attention and are seen as key determinants of environmental quality.

### Literature on globalization and environmental sustainability

The studies collectively illustrate the complex and multifaceted relationship between globalization (GN) and environmental sustainability, highlighting both positive and negative impacts.^23,24^ present contrasting views on how globalization influences environmental quality. ^23^ emphasize the composition effect, suggesting that changes in industrial structure due to globalization affect environmental outcomes. On the other hand^24^ concentrate on the scale impact, contending that increased investment and production as a result of globalization leads to increased energy consumption and CO2 emissions, which exacerbates environmental deterioration. This difference in perspective sets the stage for understanding the nuanced effects of globalization.^25^ contribute to this discussion by highlighting a different dimension: the potential for globalization to drive technological development that could mitigate environmental harm. In their opinion, environmental pressures may become more intense, but they may also be lessened by technological developments brought about by globalization.

The empirical research provides a range of results that show how globalization affects different contexts in different ways.^7^ find that globalization improves environmental quality in a broad sample of 97 countries, suggesting that globalization’s benefits might outweigh its drawbacks in some regions. Conversely^26^, and^27 ^present evidence of globalization deteriorating environmental quality, particularly in MENA countries and due to factors like low trade barriers and weak environmental governance. These findings highlight the detrimental aspects of globalization, where increased economic activity and inadequate regulations lead to environmental degradation.^28^ bridge these perspectives by exploring how renewable energy (RE) interacts with globalization. They find that while globalization exacerbates environmental degradation through the scale effect, increased use of RE can offset this impact by reducing the ecological footprint. This underscores the idea that while globalization can drive negative environmental outcomes, technological advancements and RE adoption play a crucial role in mitigating these effects.^29^ investigated the influence of globalization on carbon emissions in developing countries. Their findings also confirmed that globalization increases the environmental damage.

Together, these studies depict a complex picture where globalization’s impact on environmental sustainability depends on various factors, including industrial changes, technological progress, regulatory frameworks, and energy consumption. They illustrate that while globalization can drive economic growth and technological advancement, it also poses significant challenges to environmental quality. Thus, attaining sustainable development requires striking a balance between the advantages of globalization and the implementation of sensible environmental laws and renewable energy sources.

### Literature on natural resources and environmental sustainability

Numerous studies have explored the influence of natural resources (NR) on emissions and environmental sustainability, demonstrating a complicated link between resource exploitation and environmental deterioration. CO2 emissions are commonly used as a proxy for environmental harm in these studies. Early research, such as that by^30^, highlights the critical role of NR in sustaining long-term economic growth. The capacity of a country to effectively exploit its natural resources influences its economic trajectory and environmental outcomes.^31^ analyzed the impact of NR on CO2 emissions in five EU countries from 1985 to 2016. Their findings indicate that while trade negatively impacts the environment, the use of RE sources alongside NR can help reduce emissions. They advocate for increased adoption of RE to mitigate the environmental impact of resource exploitation.^32^ argue that human activities such as deforestation and mining lead to significant degradation of water, soil, and natural habitats, ultimately resulting in pollution and environmental damage.^33^ and^34^ discovered that NR extraction can contribute to increasing CO2 emissions and environmental deterioration.

The link between NR, economic progress, and environmental quality varies with time. Early development stages often involve high resource use and increased emissions, but as economies mature and living standards improve, there is a growing demand for greener technologies and resource conservation^35^.^36^ highlighted the importance of integrating NR management with efforts to enhance environmental quality and economic growth. Recent research provides further insight into the detrimental effects of NR on ecological sustainability. In which^37^ explored the role of *NR* in ecological sustainability among E11 countries between 1990 and 2018, finding that increased *NR*use leads to ecosystem deterioration. Similarly^38^, support these findings with their study on Mediterranean countries, reinforcing the notion that NR exploitation can compromise environmental quality. These studies collectively illustrate the dual nature of NR’s impact on the environment. While, NR are crucial for economic development and can potentially support sustainable practices when coupled with RE. But their exploitation often leads to significant environmental degradation. The evidence suggests that managing NR effectively and promoting cleaner technologies are essential for achieving both economic growth and environmental sustainability. This emphasizes the necessity of well-balanced policies that combine environmental preservation with resource management in order to lessen the negative consequences of resource extraction.

### Literature on renewable energy and environmental sustainability

The link between renewable energy (RE) and environmental sustainability is a prominent topic of research, with data repeatedly demonstrating that higher RE use contributes to enhanced environmental quality and lower CO2 emissions. Several studies affirm the beneficial impact of RE on environmental sustainability across various contexts.^39^ found that the adoption of REenhances environmental quality by lowering CO2 emissions.^40^ extended this analysis by examining the effects of various RE sources, including hydroelectricity, solar, wind, and geothermal energy, on CO2 emissions in 166 countries from 1990 to 2017. Their results underscore the collective positive influence of these RE sources on emission reductions. Similarly,^41^ investigated the effects of RE on ecological footprints in 24 OECD countries from 1980 to 2014, concluding that RE plays a crucial role in improving environmental quality.^42^ found that whereas non-renewable sources increase ecological footprints in South Asian nations, renewable energy sources decrease them. This observation lends more credence to this theory.

^43^ utilized a second-generation econometric approach to analyze the impact of RE , trade openness, and financial development on environmental issues in the 15 largest emitting economies between 1990 and 2015. Their findings highlight that RE, alongside trade openness and financial development, significantly mitigates environmental challenges.^44^ further explored the contribution of RE to ecological footprints in South Asia from 1995 to 2015, incorporating cross-sectional dependence and slope heterogeneity. Their study demonstrates that increased RE use, while maintaining current electricity production levels, contributes to reducing carbon and ecological footprints in the region.^45^ and^46^ emphasize that integrating RE technologies into national power grids can reduce reliance on fossil fuels, thereby decreasing pollutants and broadening the economy.^47^ evaluated the Environmental Kuznets Curve (EKC) hypothesis for G-7 economies using FMOLS and DOLS techniques. They found that RE utilization is linked to environmental deterioration but also supports the EKC hypothesis, suggesting a complex interplay between economic growth and environmental impact. However,^29,48^, revealed that RE use mitigates carbon emissions in Mexico and enhances environmental sustainability.

When taken as a whole, these studies demonstrate a strong link between the usage of RE and lower CO2 emissions and smaller ecological footprints. They consistently demonstrate that while RE has a positive impact on environmental sustainability, the relationship can be nuanced by other factors such as economic growth and technological advancements. According to the existing studies, increasing the usage of RE is essential to improving environmental results and lowering dependency on fossil fuels. Moreover, promoting sustainable growth and enhancing general environmental quality need incorporating RE into national energy plans.

## Theoretical framework

The STIRPAT model is used to investigate how natural resource usage and globalization affect environmental sustainability. The model was initially developed to assess the interaction between human activities and environmental repercussions. The theoretical framework of the study integrates globalization with natural resources based on STIRPAT model. The STIRPAT model in its simplest form:

1 $$\:I=P\times\:A\times\:T$$

where P stands for population, A for affluence (per capita consumption), T for technology (impact per unit of consumption), and I for the influence on the environment. We have extended the STIRPAT model by incorporating globalization and natural resources. Globalization influences environmental sustainability through changes in consumption patterns, trade, and technology transfer. To incorporate globalization, we introduce a new factor *GN*. Similarly, natural resources play a crucial role in determining environmental impacts through extraction, usage, and management. To reflect this, we introduce *NR*. The model of the study is written as follows:

2 $$\:ETS=GN\:\times\:\:NR\:\times\:\:REC\:\times\:UZ$$

The empirical model of the study can be written as:

3 $$\:{ETS}_{it}=\:{\beta\:}_{0}+\:{\beta\:}_{1}{GN}_{it}+\:{\beta\:}_{2}{NR}_{it}+\:{\beta\:}_{3}{REC}_{it}+\:{\beta\:}_{4}{UZ}_{it}+\:{\epsilon\:}_{it}$$

In the above equation, $\:ETS$ is environmental sustainability used as dependent variable measured by total greenhouse gas emissions (GHG). The $\:GN$, $\:NR$ and $\:REC$ are the independent variables known as globalization, natural resources and renewable energy consumption, respectively. However, $\:UZ$ is urbanization used as control variable. To assess environmental sustainability, the model can be used to analyze how different factors interact and contribute to environmental impacts. For example: Globalization might increase resource demand and environmental degradation through intensified trade and economic growth, but it can also lead to the adoption of cleaner technologies and international environmental agreements. Natural resources management is crucial in determining the sustainability of environmental impacts. Efficient resource use and sustainable practices can mitigate negative effects, while resource depletion and inefficient practices exacerbate them. By applying this extended STIRPAT model, researchers and policymakers can evaluate how globalization and resource use dynamics influence environmental sustainability and develop strategies to promote sustainable development.

### Data description

This study assessed the effects of GN and NR on environmental sustainability in G20 countries using a dataset from 1990 to 2020. The G20 countries (See Supplimenary table appendix A)were chosen because they account for 75% of global GHG emissions and are committed to meeting the emission reduction objectives by 2030. Therefore, to explore the impact of GN and NR on GHG emissions in G20 countries. Table 1 below provides the detailed description of the variables used in the study.

**Table 1:** Description of variables.

Variable	Abbreviation	Measurement unit	Source
Environmental sustainability	ETS	Total greenhouse gases emissions (tones thousand)	OECD
Globalization Index	GN	The globalization index comprises of economic, social, and political indices	KOF Index
Natural resources	NR	Total natural resources rents (as a % of GDP)	WDI
Renewable energy consumption	REC	It is the % of total energy consumption	IEA
Urbanization	UZ	Urban population as a % of total population	WDI

### Econometric methodology

In this section, the econometric methodology used in the study is explained step by step.

#### Cross-section dependence and slope homogeneity test

GN has increased the likelihood of cross-sectional dependency (CSD) in data collection. A number of disruptions, like oil price shocks and global financial crises, are common reasons for the spillover effects of GN. Therefore, assuming that each cross-section is homogeneous leads to inaccurate and misleading findings. To address this problem, CSD test proposed by^49^was applied in this study. Moreover, identifying the homogeneity or heterogeneity of the slopes in panel data is also critical. To meet this requirement, the heterogeneity/homogeneity of slope (HS) test was proposed by^50^. The HS test equation is as follows:

4 $$\:{\stackrel{\sim}{\varDelta\:}}_{SCH}\:=\:{\left(N\right)}^{\raisebox{1ex}{$1$}\!\left/\:\!\raisebox{-1ex}{$2$}\right.}\:{\left(2k\right)}^{-\raisebox{1ex}{$1$}\!\left/\:\!\raisebox{-1ex}{$2$}\right.}\:\left(\frac{1}{N}\stackrel{\sim}{S}-k\right)$$

The CSD equations are given below:

5 $$\:CD=T{\sum\:}_{i=1}^{N-1}{\sum\:}_{j=i+1}^{N}{\widehat{\rho\:}}_{ij}^{2}$$

6 $$\bf \it CD = \sqrt{\frac{2T}{N(N-1)}} (\sum_{i=1}^{N-1}\sum_{j=i+1}^{N}{\widehat{\rho\:}}_{ij}$$

#### Panel unit root tests

In the presence of the CSD issue, the first-generation stationary test yields conflicting findings. Therefore, after confirming the existence of CSD in panel data, the study employs a second-generation stationary test of cross-sectional augmented IPS (CIPS) developed by^51^ to check the presence of a unit root in the data set.

7 $$\:{\varDelta\:CA}_{i,t}=\:{\varphi\:}_{i}+\:{\varphi\:}_{i}{Z}_{i,t-1}+{\varphi\:}_{i}{\stackrel{-}{CA}}_{t-1}+\:{\sum\:}_{I=0}^{p}{\varphi\:}_{iI}{\Delta\:}{\stackrel{-}{CA}}_{t-1}+\:{\sum\:}_{I=0}^{p}{\varphi\:}_{iI}{\Delta\:}{CA}_{i,t-1}+\:{\mu\:}_{it}$$

Where $\:{\stackrel{-}{CA}}_{t-1}$ and $\:\varDelta\:{\stackrel{-}{CA}}_{t-1}$ are the averages of all cross-sectional observations.

8 $$\:\widehat{CIPS}=\:\frac{1}{N}\:{\sum\:}_{i=1}^{n}{CDF}_{i}$$

In the above equation, CADF is a cross-sectional augmented Dickey-Fuller.

#### Panel cointegration test

Examining the integration order of the variables is the first step in testing for cointegration between them. Because to CSD and heteroscedasticity in the data, the first- and second-generation panel cointegration tests are also unsuccessful. The^52^ panel cointegration test is used to resolve the structural break, autocorrelation, and CSD concerns:

9 $$\:{LM}_{\tau\:}=\:\raisebox{1ex}{${\widehat{\psi\:}}_{i}$}\!\left/\:\!\raisebox{-1ex}{$SE\:\left({\widehat{\psi\:}}_{i}\right)$}\right.$$

10 $$\:{LM}_{\psi\:}=T{\widehat{\psi\:}}_{i}\left(\raisebox{1ex}{${\widehat{\omega\:}}_{i}$}\!\left/\:\!\raisebox{-1ex}{${\widehat{\phi\:}}_{i}$}\right.\right)$$

#### Long run estimation

After obtaining the necessary information for long-run parameter estimation, this study used the continuously updated fully modified (CUP-FM) approach first presented by^53^as well as the continuously updated bias-corrected (CUP-BC) method. CUP-FM yields more accurate findings than other well-known long-run estimation methods such the fully modified (FMOLS) and generalized method of moments (GMM). In the face of several panel data econometric problems, including CSD, heteroscedasticity, autocorrelation, and endogeneity, it produces reliable and effective findings even with small data sets^54^. Moreover, it has been believed that this strategy involves continually adding the error term after the common latent components until convergence is achieved. It is mathematically written as:

11 $$\:\widehat{\beta\:}cup,\:\widehat{F}cup=argmin\:\frac{1}{n{T}^{2}}\:{\sum\:}_{i=1}^{n}{\left({y}_{i}-\:{x}_{i}\beta\:\right)}^{{\prime\:}}{M}_{F}\left({y}_{i}-\:{x}_{i}\beta\:\right)$$

Because these sophisticated panel data estimate techniques are successful at resolving the main issues associated with evaluating data from G20 nations, the study uses them, including CUP-FM and CUP-BC methods. These methods are particularly suited for dealing with cross-sectional dependence, where economic and environmental events in one country can significantly impact others, reflecting the interconnected nature of G20 economies. Additionally, they account for heterogeneity across countries, allowing for a nuanced analysis that recognizes the differing economic structures, income levels, and policy environments within the G20. The methods are also designed to estimate long-run relationships, which is crucial given the 30-year period of the study, enabling a reliable assessment of the enduring effects of globalization, natural resource use, renewable energy consumption, and urbanization on GHG emissions. Additionally, endogeneity is corrected for using CUP-FM and CUP-BC, lowering the possibility of bias and guaranteeing that the findings accurately reflect causal connections. The study’s dependable findings, which are essential for guiding policy decisions targeted at improving environmental sustainability, are produced by employing these strong methodologies.

## Empirical findings

The descriptive statistics for all variables for the whole sample of G20 nations, including high- and middle-income nations are shown in Table 2. The G20 high-income and middle-income nations differ significantly from one another, as seen by the descriptive data. This also show greater levels of globalization, renewable energy consumption, and GHG emissions in the high-income countries. The utilization of resources and urbanization are more variable in middle-income nations, and there are notable disparities in the use of renewable energy sources and the exploitation of natural resources. Comprehending these figures facilitates the customization of policies that target the distinct requirements and obstacles faced by every G20 income category.

**Table 2:** Descriptive statistics.

Variable	Obs.	Mean	Max	Min	St.dev
G20 countries					
ETS	600	1.577	5.139	0.140	1.040
GN	600	2.152	3.990	−1.272	1.233
NR	600	3.341	4.362	1.744	0.573
REC	600	0.203	1.637	−1.965	0.763
UZ	600	2.878	5.794	−0.962	1.566

**High-income G20 countries**					
ETS	330	0.606	1.799	0.033	0.415
GN	330	3.047	4.362	1.674	0.660
NR	330	2.205	4.497	1.272	1.167
REC	330	0.328	1.367	−1.965	0.687
UZ	330	2.697	5.794	−1.367	1.158

**Middle-income G20 countries**					
ETS	270	0.099	1.799	0.098	0.670
GN	270	3.130	4.295	1.710	0.662
NR	270	1.769	4.497	−1.272	1.479
REC	270	0.248	1.531	−1.730	0.657
UZ	270	2.341	4.852	−1.367	2.139

Policymakers are better able to explain the many environmental externalities connected to the variables and, as a consequence, create well-organized policies when SH and CSD concerns are included in the panel data. Table 3 presents the results of the CSD test and rejects the null hypothesis of no CSD at a significance level of 1%. This shows that there is a substantial CSD between the investigated variables in each of the three G20 country samples. Emissions in one country are related to those in other countries within the same group. Globalization levels are correlated across countries in the same group. The use of natural resources shows strong cross-sectional dependence, reflecting regional or global patterns of resource exploitation. The usage of renewable energy is also correlated across countries, suggesting shared trends or policies. Urbanization levels show significant cross-sectional dependence, indicating that urban growth patterns in one country are related to those in other countries within the same group.

**Table 3:** Pesaran (2007) Cross-sectional dependence test results.

Variable	CSD test statistic		
	G20 countries	High-income G20 countries	Middle-income G20 countries
ETS	43.41***	37.37***	60.59***
GN	29.25***	45.02***	62.26***
NR	91.99***	23.80***	40.87***
REC	95.12***	18.28***	28.40***
UZ	55.98***	41.52***	62.23***
Note: *** significant at 1% level			

The findings of the delta tests performed to check for slope homogeneity are displayed in Table 4. The findings demonstrate that slope heterogeneity exists in all three samples of G20 countries and that the presence of slope homogeneity is rejected as a null hypothesis at the 1% level of significance. In case of overall G20 countries, there is substantial variation in the relationships between the variables across different countries, suggesting that a one-size-fits-all model might not be appropriate. Policies or models should consider this heterogeneity to be more effective. The substantial slope heterogeneity suggests that high-income nations react differently to the factors under investigation, necessitating the customization of interventions or policies. The results indicate significant variability in how the variables affect GHG emissions and other factors, suggesting that middle-income countries also require targeted approaches to address their unique circumstances. These findings emphasize the need of taking into consideration cross-country variations in econometric modeling and policy development in order to more fully comprehend and resolve environmental and economic concerns in the G20 nations.

**Table 4:** Pesaran & Yamagata (2008) Slope homogeneity test results.

	SH test statistic		
	G20 countries	High-income G20 countries	Middle-income G20 countries
Δ	−4.519***	−3.238***	−3.290***
Δ_adj_	−5.180***	−5.258***	−5.698***
Note: *** significant at 1% level			

Second-generation panel unit root tests must be used to verify the order of variable integration in the presence of heterogeneity and CSD. Table 5 displays the CIPS unit root test results for each of the three G20 country samples. The findings of the CIPS unit root test for each of the three G20 country samples revealed that all of the variables (ETS, GN, NR, REC, and UZ) are I (1), rejecting the null hypothesis. This finding makes it easier to select suitable panel estimation techniques for analyzing the long-term relationships between the variables.

**Table 5:** Pesaran (2007) CIPS unit roots test results.

Variable	G20 countries		G20 High-income countries		G20 Middle-income countries	
	Level	1st Diff	Level	1st Diff	Level	1st Diff
ETS	−1.953	−5.333**	−1.447	−4.184**	−1.663	−4.376**
GN	−0.460	−5.489**	−0.522	−6.190**	−0.953	−2.333**
NR	−1.002	−3.892**	−1.594	−6.167**	−1.120	−3.190**
REC	−1.200	−6.190**	−1.618	−4.190**	−1.585	−4.993**
UZ	−1.869	−5.511**	−0.522	−5.291**	−1.460	−3.489**
Note: ** significant at 5% level						

A structural break in economics refers to a significant change or significant revisions to the economy, politics, or technology that results in a long-lasting shift in a pattern. The main advantage of the^52^cointegration method is that it supports the long-term association while taking into account the structural break problem. The outcomes of the^52^ cointegration test are shown in Table 6. The results confirm the existence of cointegration in all three samples of G20 countries. In summary, the results suggest that there is generally strong evidence of cointegration among the variables for G20 countries overall, with varying robustness in high-income and middle-income countries. For high-income G20 countries, the cointegration evidence is strongest in the absence of structural shifts, while for middle-income countries, the cointegration relationship is affected by both mean and regime shifts. This indicates that while long-term relationships exist, they are influenced by changes in economic conditions, particularly in middle-income countries.

**Table 6:** Westerlund & Edgerton (2008) cointegration test results.

Model	No shift	Mean shift	Regime shift
G20 countries			
LMι	4.299**	4.910**	5.176**
LM_Ψ_	5.583**	5.068**	5.413**

**High-income G20 countries**			
LM_ι_	−4.330**	−2.371**	−2.241**
LM_Ψ_	−3.681**	−1.487**	−1.755**

**Middle-income G20 countries**			
LM_ι_	6.131**	−4.171**	−4.110**
LM_Ψ_	5.151**	−3.182**	−3.571**
Note: ** significant at 5% level			

After establishing the existence of cointegration, the next step is to employ the CUP-FM and CUP-BC estimation methods to examine the long-run coefficients and the results are shown in Table 7. The findings of CUP-FM suggest that $\:GN$ significantly increases GHG emissions, reducing environmental sustainability in G20 countries. An increase in $\:GN$leads to increase 0.137% of emissions in the environment. This is because GN stimulates economic activity, which expands transportation services, resulting in a scale impact and, ultimately, low environmental sustainability. Several subsequent studies, including^24,26,27,55^, have supported similar findings. G20 nations are suffering as a result of GN, which has contributed to deforestation and the widespread use of fossil fuels and natural resources^36^. Furthermore, its strong emphasis on trade, particularly imports and exports to G20 nations, contributes to environmental degradation^56^.

Similarly, $\:NR$ has a positive coefficient of 0.232, indicating that increasing the $\:NR$will result in greater emissions. Many recent researches, including^34,37,57,58^supports this link. The significant positive association among natural resource use and GHG emissions indicates that extracting and processing natural resources (e.g., mining, drilling) are energy-intensive activities that typically result in substantial GHG emissions. Many natural resources, such as fossil fuels, are used for energy production, directly contributing to GHG emissions^59,60^.

The $\:REC$, on the other hand, shows a negative and significant coefficient of 0.719 at a 1% level of significance, demonstrating that excessive use of $\:REC$will help to lower emission levels in G20 countries, and studies by^61–65^ found the same results. On the other hand, from 2015 to 2020, the G20 raised its average share of renewables in its entire mix of primary energy supply by 32%, reaching 7% in that year. However, by rapidly phase-out of fossil fuels and promoting development in $\:REC$, significant reductions in emissions from the energy sector may be attained. The negative and significant impact of renewable energy on GHG emissions highlights the benefits of shifting to cleaner energy sources. Renewable energy sources (e.g., wind, solar) produce electricity with little to no GHG emissions compared to fossil fuels. Increasing the share of renewables in the energy mix reduces reliance on fossil fuels, leading to lower overall emissions^66–70^. High-income countries often have more resources and infrastructure to invest in renewable energy, resulting in a significant reduction in emissions as they transition to cleaner energy sources. However, in middle-income G20 countries, although the impact is smaller, renewable energy still plays a crucial role in reducing emissions. However, these countries might have less capacity for rapid adoption of renewable technologies compared to high-income nations.

The positive connection among urbanization and GHG emissions suggests that urban areas typically have higher energy demands for buildings, transportation, and infrastructure, leading to increased emissions and consistent with the findings of^9^. Urbanization often correlates with higher consumption levels and increased use of energy-intensive goods and services. In high-income G20 countries, the impact is moderate, likely due to advanced urban infrastructure and energy efficiency measures that partially offset the emissions from increased urban activities. In middle-income G20 countries, urbanization has a stronger impact on GHG emissions in these countries as they often undergo rapid and unplanned urban growth, which can lead to inefficiencies and higher emissions. Furthermore, the CUP-BC test findings complement the CUP-FM analysis results, confirming the linkages between $\:GN,\:NR,\:REC,\:UZ\:$and $\:ETS$.

**Table 7:** Bai & Kao (2006) CUP-FM and CUP-BC test results.

Variable	G20 countries		G20 High-income countries		G20 Middle-income countries	
	CUP-FM	CUP-BC	CUP-FM	CUP-BC	CUP-FM	CUP-BC
GN	0.137***	0.013**	0.188***	1.479**	0.122***	0.256***
NR	0.232**	0.018***	0.211*	0.479**	0.295***	0.279***
REC	−0.719***	−2.105***	−0.405***	−0.019***	−0.037**	−0.125***
UZ	0.369***	0.006***	0.019**	0.405***	0.671***	0.371**
Note: *, **, and *** denote significance at the 10%, 5%, and 1% levels.						

## Discussion of the results

Increased international trade and economic integration frequently result in higher emissions as confirmed by the positive and considerable influence of globalization on GHG emissions in G20 countries. This can be due to several factors, including that it boosts trade and economic activity, leading to higher production and consumption, which in turn increases energy use and emissions. Globalization can lead to the relocation of high-emission industries to countries with less stringent environmental regulations. Greater international trade requires more transportation, contributing to higher emissions from shipping, aviation, and road transport. The stronger positive effect in high-income countries may be due to their significant role in global trade and higher consumption levels, which are often associated with more energy-intensive lifestyles and greater emissions. While the impact is still positive, it may be less pronounced compared to high-income countries. Industrialization and urbanization are common processes in middle-income nations, and they may both raise emissions, albeit at different rates.

The bulk of GHG emissions in the G20 are produced by the transportation, industrial, and power generating sectors. To achieve net zero CO2 emissions by about 2050 and quickly reduce all GHG emissions after that, it is imperative that all sectors undergo change. This includes maintaining and growing vital global carbon sinks like forests. This is a result of the G20 continuing to fund the fossil fuel industry with promises of USD 298 billion between 2020 and 2021, almost the same amount as the G20’s whole green recovery pledge. Furthermore, these subsidies violate commitments of G20 to reduce inefficient fossil fuel subsidies. However, the impact of $\:NR$ on $\:ETS$ in high-income G20 countries is positive, but the intensity of the impact is low as compared to middle-income countries because in these countries, a carbon price currently covers nearly half of all CO2 emissions connected to energy. In the past several years, the majority of nations have expanded or implemented carbon taxes or emissions trading schemes. However, in order to meet their long-term emission control goals, middle-income G20 nations must employ a wide range of policy instruments. The high-income G20 countries often have advanced technologies but also high resource consumption, which means they may still have significant emissions due to resource-intensive activities and industries. However, middle-income G20 countries might be experiencing rapid industrialization and resource exploitation, leading to a high impact on GHG emissions as they develop their economies and infrastructure.

In conclusion, increasing production, consumption, and resource exploitation brought about by globalization and the usage of natural resources often result in higher greenhouse gas emissions. The renewable energy sources or consumption offer cleaner energy options and they considerably lower greenhouse gas emissions. Because of the greater energy demand and consumption in metropolitan areas, urbanization generally results in increased emissions. These findings highlight how crucial it is to address each of these variables through focused policies and programs in order to lessen their influence on environmental sustainability.

## Conclusion

The research uses data from 1990 to 2020 to examine how globalization and natural resources affect the sustainability of the environment in G20 nations. Once CSD and slope homogeneity have been confirmed, the study employs second-generation panel estimate methods. The order of variable integration is verified using the CIPS unit root test. To check the cointegration, the second-generation^52^ cointegration test is applied, and CUP-FM and CUP-BC econometric approaches are used to estimate long-run coefficients. The study’s findings indicate that the model has SH and CSD, and the order of variable integration at I(1) is validated by the CIPS unit root test. The CUP-FM and CUP-BC methods’ long-term estimation results demonstrated that natural resources and globalization reduce environmental sustainability by raising GHG emissions in G20 nations. The results of heterogeneity study in high- and moderate-income G20 nations are likewise comparable.

This research offers fresh insights and insightful solutions to address environmental problems. It is crucial to emphasize that while calculating environmental sustainability, natural resources and globalization should be taken into account in addition to elements like economic expansion, urbanization, and the use of fossil fuels for energy. The results indicate that the primary reason for the rise in emissions in the G20 countries is the energy consumption through fossil fuels. Which is directly linked to the use of fuel for transportation in the fields of aviation, shipping, and road transportation. In addition, the G20 has continued to heavily subsidize the fossil fuel industry in recent years, despite earlier pledges to eliminate these inefficient forms of support. Compared to high-income countries, the effect is more noticeable in middle-income nations. However, it is predicted that by reducing GHG emissions, adopting renewable sources will be better for the environment. To accomplish such targets, these nations should formally pledge to cut their emissions by putting emission-reducing measures into place.

### Policy implications

The study’s conclusions offer some recommendations for G20 policy objectives. These nations should embed environmental sustainability clauses in trade agreements to ensure that globalization does not undermine environmental goals. In addition, promote trade practices that support the use of sustainable resources and clean technologies. Similarly, support the development of green trade policies that incentivize the production and consumption of environmentally friendly goods and services. Moreover, implement policies that encourage the sustainable use of natural resources. This includes adopting practices that reduce resource extraction and waste, and investing in technologies that enhance resource efficiency. Above all, reassess and phase out subsidies for fossil fuels, which continue to contribute to high GHG emissions. Redirect these subsidies towards renewable energy sources and energy efficiency measures. Surge investments and subsidies for renewable energy sources (e.g., solar, wind, and hydropower) to reduce reliance on fossil fuels. Also, ensure policies are in place to support the transition to cleaner energy technologies. By applying these policies, G20 countries can address the challenges identified in your study, reduce GHG emissions, and enhance environmental sustainability. It is crucial that these efforts are supported by robust monitoring and evaluation mechanisms to ensure effectiveness and adapt to changing environments.

### Future research

Further investigation could expand on this study by exploring several key areas to deepen the understanding of how globalization and natural resources affect environmental sustainability in G20 countries. Furthermore, detailed assessments of emerging technologies and their potential to reduce emissions, along with economic modeling to predict the effects of various interventions, would provide further insights. This study includes the G20 countries, which are prominent both economically and geographically. A future study should look at the impact of pre- and post-globalization on environmental sustainability in the OECD, G7, and BRICS countries. Impending studies may also determine how each facet of globalization influences environmental sustainability, assisting governments in developing comprehensive environmental policies. Finally, the development of advanced econometric techniques can offer more precise analyses of these complex relations.

## Electronic supplementary material

Below is the link to the electronic supplementary material.

Supplementary Material 1

## Data Availability

The datasets analyzed during the current study are available at World Bank database (WDI) https://data.worldbank.org/ , the OECD data repository https://data.oecd.org/ , and the IEA repository https://www.iea.nl/data-tools/repository .

## Abbreviations

Carbon emissions: CO2
Continuously Updated Fully Modified and Continuously Updated Bias-Corrected: CUP-FM & CUP-BC
Cross-sectional dependence: CSD
Environmental sustainability: ETS
Globalization: GN
Greenhouse gas emissions: GHG
Natural resources: NR
Renewable energy consumption: REC
Slope homogeneity: SH
Urbanization: UZ
