# Supplementary material for: Examining the impact of globalization and natural resources on environmental sustainability in G20 countries
Source: Sci Rep. 2024 Dec 28;14:30921. doi: 10.1038/s41598-024-81613-6 (PMC11680794; doi:10.1038/s41598-024-81613-6)
Supplement: Supplementary file 1 — Supplementary Material 1 [file 41598_2024_81613_MOESM1_ESM.docx]

**Appendix A**

**List of G20 Countries**

| **G20 Countries** | **High-income G20 countries** | **Middle-income G20 countries** |
| --- | --- | --- |
| Argentina | Australia | Argentina |
| Australia | Canada | Brazil |
| Brazil | EU | China |
| Canada | France | India |
| China | Germany | Indonesia |
| EU | Italy | Mexico |
| Germany | Japan | Russia |
| France | South Korea | South Africa |
| India | Saudi Arabia | Turkiye |
| Indonesia | United Kingdom |  |
| Italy | United States of America |  |
| Japan |  |  |
| Mexico |  |  |
| Russia |  |  |
| Saudi Arabia |  |  |
| South Africa |  |  |
| South Korea |  |  |
| Turkiye |  |  |
| United Kingdom |  |  |
| United States of America |  |  |
